# Novel surfactant stabilized PLGA cisplatin nanoparticles for drug delivery applications

**DOI:** 10.3906/kim-2105-41

**Published:** 2021-07-06

**Authors:** Samra UMAR, Manzar ZAHRA, Mohammad Salim AKHTER, Amir WASEEM

**Affiliations:** 1 Department of Chemistry, Quaid-i-Azam University, Islamabad, Pakistan; 2 Department of Chemistry, Lahore Garrison University, Lahore, Pakistan; 3 Department of Chemistry, College of Science, University of Bahrain, Sakhir, Bahrain

**Keywords:** Cisplatin, surfactants, drug delivery, PLGA nanoparticles

## Abstract

In the current study, cisplatin loaded (Poly(lactic-co-glycolicacid)) (PLGA) stabilized with 4-phenylphenacylbromide based surfactants nanoparticles were developed for drug delivery applications. For the course of this study four new 4-phenylphenacylbromide based compounds abbreviated as PA(C_2_)_3_, PA(C_8_)_3_, PAC_16_ and PAC_18_ have been synthesized by the reaction of 4-phenylphenacylbromide with various long chain amines. The new surfactants were characterized with ^1^H and ^13^C NMR and FTIR spectroscopy. The critical micelle concentration (CMC) of newly synthesized surfactants are in the range of 0.024–0.091 mM. The newly synthesized surfactants have been incorporated in PLGA based nanoparticles and are used for drug encapsulation and delivery of cisplatin. The drug encapsulation efficiency for newly fabricated nanoparticles was found to be in the range of 65 ± 1.5 to 67 ± 1.6%, the drug loading was observed in the range of 1.96 ± 0.11 to 2.31 ± 0.19%, whereas the maximum drug release was found to be 85.1 ± 1.1%.

## 1. Introduction

Surfactants are a very diverse class of amphiphilic molecules that find applications in almost every field of life. Their innovative nature and striking properties have always been a source of attraction for chemists and biochemists. Surfactants can be obtained from various sources; they can be naturally occurring or synthetic. The naturally occurring surfactants include glycerol-based lipids, which are vital components of cell membranes. Historically, the soaps were the first type of surfactants that were discovered, manufactured, and used by humans [1]. All detergents, wetting agents, emulsifiers, foaming agents, corrosion inhibitors, antistatic agents etc. are usually surfactants [2,3 ]. The first surfactants discovered in living systems were pulmonary surfactants, which have an important role in lowering the surface tension in alveoli in lungs. From detergents to cosmetics, surfactants come in handy for human use in every possible way. Their surface-active properties and the tendency to alter surface tension of the medium make them a unique and diversely applicable class of compounds [4–6].

Poly(D,L-lactic-co-glycolic acid) (PLGA) is a copolymer of poly(lactic acid) (PLA) and poly(glycolic acid) (PGA). It is been explored by many investigators for developing nanoparticles (NPs) for drug delivery (DD) applications for cancer diagnosis and therapy due to its high biocompatibility and biodegradability [7, 8 ].

Temperature-sensitive, targetable cisplatin nanocarriers based on poly(propylene succinate) copolymers with poly(ethyleneglycol) (PPSu-PEG) were prepared and evaluated in vitro for their potential for a more selective delivery of cisplatin to tumours using local hyperthermia was reported earlier. One-pot melt-polymerization under vacuum was used to prepare the copolymer and loaded with cisplatin using double emulsion method [9].

In a recent study, folate-poly(ethylene glycol)-poly(propylene succinate) nanoparticles (FA-PPSu-PEG-NPs) were developed and used as a vehicle for targeted delivery of the anticancer drug paclitaxel in breast and cervical cancer cell lines. FA-PPSu-PEG-NPs can also be used as vehicles for other anticancer drugs [10]. The drug release profile shows a biphasic nature, with a rapid release during the first 24 h, followed by a prolonged release phase, reaching a plateau after 96 h.

It is common to produce PLGA NPs through nanoprecipitation method, also called the solvent-evaporation or solvent-switch method [11]. Using this technique, usually NPs sizes between 100 and 200 nm is obtained depending on the solvent used, solvent ratio and polymer-drug concentration. It offers a good reproducibility and particle stability [7,12 ]. Despite these advantages, the stability of NPs to some extent becomes limited when polymeric NPs are loaded with drugs. One way to increase the NPs colloidal stability is the use of amphiphilic substances called surfactants. Different investigators used range of surfactants from nonionic to ionic (cationic or anionic) [13, 14 ]. In a recent study [15] different surfactant, i.e. PVA, Pluronic F68, Pluronic F127 and polysorbates (Tween 20, Tween 80) were used in the preparation of PLGA NPs loaded with protein kinase C inhibitor and the result shows the drug encapsulation efficiency varied from 31 to 75% with a drug loading of 1.3%–2% and partially hydrolyzed PVA was the surfactant of choice. Similarly in another recent study [16], positively charged curcumin nanoparticles were synthesized using PLGA and cationic surfactant cetyltrimethylammonium bromide (CTAB) and was investigated as fungicidal agents.

In the present communication, four new 4-phenylphenacylbromide based surfactants were synthesized by reacting with long chain amines and used as stabilizing the cisplatin loaded PLGA for DD applications.

## 2. Experimental

### 2.1 Chemicals

Chemicals like 4-phenyl phenacylbromide, triethyl amine, trioctyl amine, hexadecyl amine, octadecyl amine, ethylbromide, cisplatin (CP), *o*-phenylenediamine (OPDA) and solvents like methanol, ethanol, chloroform, acetone, DMSO, DMF, HCl, THF used in this research work were purchased from Sigma Aldrich. Deionized water was used throughout the study. PLGA, cis-platin and *o*-phenylenediamine used for the drug delivery application were also purchased from Sigma Aldrich. All solutions were freshly prepared and used immediately.

### 2.2 Instrumentation

^1^H-NMR and ^13^C-NMR analysis of synthesized surfactants were done at 300 MHz using nuclear magnetic resonance (NMR) spectrometer (Bruker) with a 5 mm PROBHD BBO BB-1H probe for ^1^H and ^13^C NMR at 295 K. Deuterated chloroform (CDCl_3_) and Dimethyl sulfoxide (DMSO) were used as a solvent. FT-IR spectra of synthesized surfactants were done on BRUKER-TENSOR-27 in the range of 4000 cm^−1^ to 400 cm^−1^ (resolution was 1 cm^−1^ and 15 scan) to get insight about the functional groups and structural composition of surfactants. Melting points were recorded on a capillary tube using electro thermal melting apparatus, model MPD Mitamura (Japan).

### 2.3 Synthesis

The compounds in this series were synthesized by the reaction of 4-phenyl phenacyl bromide using amines such as triethyl amine, trioctyl amine, hexadecyl amine, octadecyl amine.

#### 2.3.1 Synthesis of PA(C_2_)_3_

**Scheme 1:**
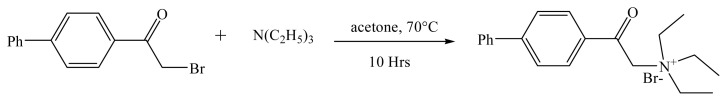
Reaction scheme shows the synthesis of PA(C_2_)_3_.

One gram of 4-phenyl phenacyl bromide was dissolved in 100 mL dry acetone and the resulting solution was transferred to a 250 mL two neck round bottomed flask. The solution was heated up to 70 °C and 0.5 mL of triethylamine was added from a dropping funnel with constant stirring. The reaction mixture was refluxed for 10 h maintaining the reaction conditions. The chemical equation for the reaction is given in Scheme 1.

The resulting brownish precipitates of the cationic surfactant were filtered and washed with ethyl acetate-hexane mixture several times, dried and collected as amorphous brown solid.

#### 2.3.2 Synthesis of PA(C_8_)_3_

**Scheme 2:**
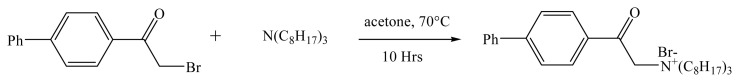
Reaction scheme shows the synthesis of PA(C_8_)_3_.

One gram of 4-phenyl phenacyl bromide was dissolved in 100 mL dry acetone and the resulting solution was transferred to a 250mL two neck round bottom flask. The solution was heated up to 70 °C and 1.6 mL of trioctylamine was added from a dropping funnel with constant stirring. The reaction mixture was refluxed for 10 h maintaining the reaction conditions. The chemical equation for the reaction is given in Scheme 2.

The brown precipitates of cationic long chain surfactant were filtered and washed with ethyl acetate-hexane mixture, dried and collected.

#### 2.3.3 Synthesis of PAC_16_

**Scheme 3:**
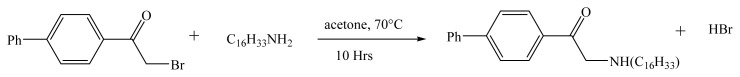
Reaction scheme shows the synthesis of PAC_16_.

One gram of 4-phenyl phenacyl bromide and 0.86 g of hexadecyl amine were dissolved in 100 mL dry acetone and the resulting mixture was transferred to a 250mL two neck round bottom flask. The contents were heated up to 70 °C with constant stirring. The reaction mixture was refluxed for 10 h maintaining the reaction conditions. The chemical equation for the reaction is given in Scheme 3.

The white precipitates of neutral compound were filtered and washed with ethyl acetate-hexane mixture, dried and collected and melting point of the product was calculated.

#### 2.3.4 Synthesis of PAC_18_

**Scheme 4:**
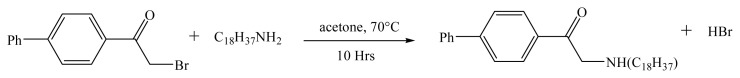
Reaction scheme shows the synthesis of PAC18.

One gram of 4-phenyl phenacyl bromide and 0.97 g of octadecyl amine were dissolved in 100mL dry acetone and the resulting mixture was transferred to a 250mL two neck round bottom flask. The contents were heated up to 70 °C with constant stirring. The reaction mixture was refluxed for 10 h maintaining the reaction conditions. The chemical equation for the reaction is given in Scheme 4.

The neutral product was obtained as white solid and was filtered and washed with ethylacetate-hexane mixture, dried and collected and its melting point was recorded.

### 2.4 CMC calculations

For this study, the CMC of synthesized surfactants has been determined by tensiometric method. 0.001, 0.005, 0.010, 0.015, 0.020, 0.025, 0.030, 0.035, 0.040, 0.045, 0.050 mM dilutions for each surfactant were prepared from 1 mM stock solution. The value of surface tension for each dilution was measured by placing 30 mL of each dilution in the tensiometer container one by one. These values were then plotted as a function of concentration of the surfactant in the solution and CMC was identified from the graph as the point where the decreasing slope and baseline of minimal surface tension values intersected.

### 2.5 Drug encapsulation in PLGA NPs stabilized with surfactants

The novel surfactants have been used for the drug delivery application of cis-platin, a well-known anticancer drug. The drug is encapsulated with surfactant by nanoprecipitation method, using PLGA as a particle forming agent. The added surfactant from the solution plays a role in stabilization PLGA nanoparticles. PLGA NPs were prepared by a nanoprecipitation method as previously described [17,18 ] with slight modifications, in brief, 5 mg cis-platin drug and 100 mg PLGA were accurately weighed and added to a test tube. A total of 0.2 mL DMSO and 1.8 mL acetone was added to the test tube and the contents were sonicated for 1 min to get a homogeneous mixture. The resulting organic mixture was added to a 250mL beaker containing 10 mg surfactant solution in 20 mL deionized water under constant stirring. Visible nanoprecipitation was seen to occur. The mixture was stirred for 3 h at room temperature to evaporate organic solvent. Later, the colloidal mixture was centrifuged at 14000 rpm, 1600 g for 15 min to separate nanoparticles from supernatant. The supernatant was decanted, and nanoparticles were washed with deionized water and lyophilized. The set of prepared nanoparticles and their theoretical composition is shown in Table 1.

### 2.6 Spectrophotometric determination of drug encapsulation and release study

The quantification of cisplatin was carried out by a modification of UV-Vis spectrophotometric method (o-phenylenediamine; OPDA-derivatization) previously reported [19]. The determination of drug content is based on the absorbance measurement of the reaction product of released cis-platin and OPDA. The product was obtained in 10^−5^ M HCl at 90 °C in 30 min. The percentage of entrapped cisplatin (drug encapsulation efficiency) was measured by dissolving a dried pellet of known weight with 100μL of DMF by vortexing for 30min. For drug release study, the encapsulated cis-platin was released by suspending all sets of prepared nanoparticles in phosphate buffer saline at pH 7.4 one by one for several hours. The mixture was centrifuged at 14000 rpm for 15 min and the resulting supernatant was checked for the presence of cisplatin by UV-Vis absorbance around 710 nm.

## 3. Results and discussion

### 3.1 Schematic structure of synthesized surfactants

**Figure 1:**
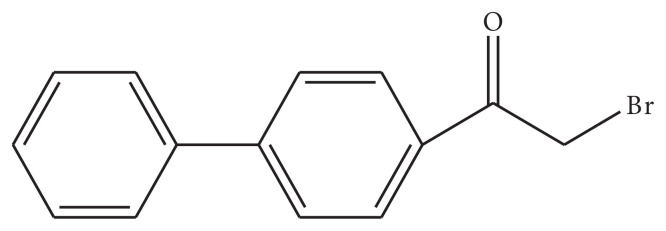
Structure of 4-phenyl phenacylbromide.

**Figure 2:**
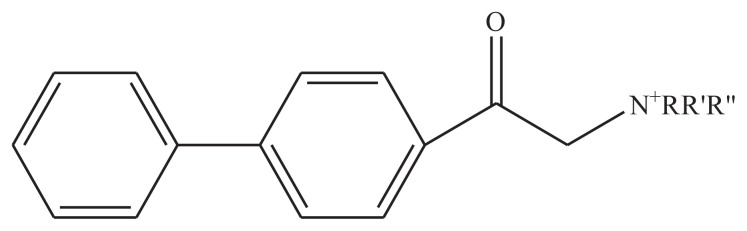
Schematic structure of newly synthesized compounds where, R= C_2_H_5_, R′= C_2_H_5_, R″= C_2_H_5_ for **PA(C_2_)_3_** R= C_8_H_17_, R′= C_8_H_17_, R″= C_8_H_17_ for **PA(C_8_)_3_** R= C_16_H_33_, R’= H for **PAC_16_**. R= C_18_H_37_, R’= H for **PAC_18_**

Four new 4-phenyl phenacylbromide based compounds have been synthesized by reacting the precursor with different long chain tertiary and primary amines and three of these compounds are regarded as surfactants. These surfactants are used in the drug delivery application of cis-platin along with PLGA. The structure of 4-phenyl phenacylbromide and the synthesized surfactants are shown in Figures 1 and 2. The physical data of these surfactants are summed up in the Table 2.

### 3.2 Characterization

#### 3.2.1 Characterization by physical parameters

The newly synthesized series can be characterized in terms of physical parameters such as physical state, color, melting point etc., as illustrated in Table 3. All newly synthesized compounds are solids at room temperature and have sharp melting points. All of these shows moderate solubility in common organic solvents as well as in water.

#### 3.2.2 Characterization by spectroscopic data

All new synthesized 4-phenylphencylbromide based compounds have been characterized by ^1^H and ^13^C NMR spectroscopy and FTIR spectroscopy. The successful synthesis of all 4 surfactants has been confirmed by spectroscopic techniques.

#### 3.2.3 ^1^H NMR Spectroscopy

**Figure 3:**
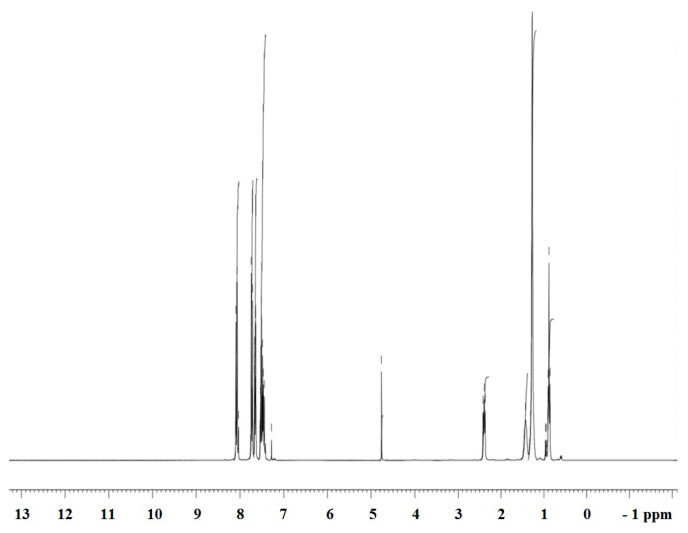
^1^H NMR spectrum of PA(C_8_)_3_ surfactant.

The ^1^H NMR spectrum was recorded for all 4-phenyl phenacylbromide based compounds. The spectral data is found to be in accordance with the predicted structure hence it can be said that the synthesis of new surfactants was successful. The ^1^H NMR spectrum of PA(C_8_)_3_ is shown in Figure 3. The characteristic peaks and their multiplicity are listed in Table 4. ^1^H-NMR spectra of novel synthesized compounds (PA(C_2_)_3_, PA(C_8_)_3_, PAC_16_ and PAC_18_) show characteristics chemical shifts: δ (ppm): 7.42–8.07 (m), 7.44–8.09 (m), 7.42–7.92 (m) and 7.42–8.02 (m) for aromatic-H respectively, 4.50 (s), 4.76 (s), 4.50 (s) and 4.56 (s) for CH_2_ respectively, 2.38 (q), 2.39 (t), 2.36 (m) and 2.34 (m) for CH_2_-N respectively, 1.27 (m), 1.33 (m) and 1.33 (m) for long chain respectively, and 1.25 (t), 0.90 (m), 0.96 (m) and 0.97 (m) for CH_3_ respectively.

#### 3.2.4 ^13^C NMR spectroscopy

**Figure 4:**
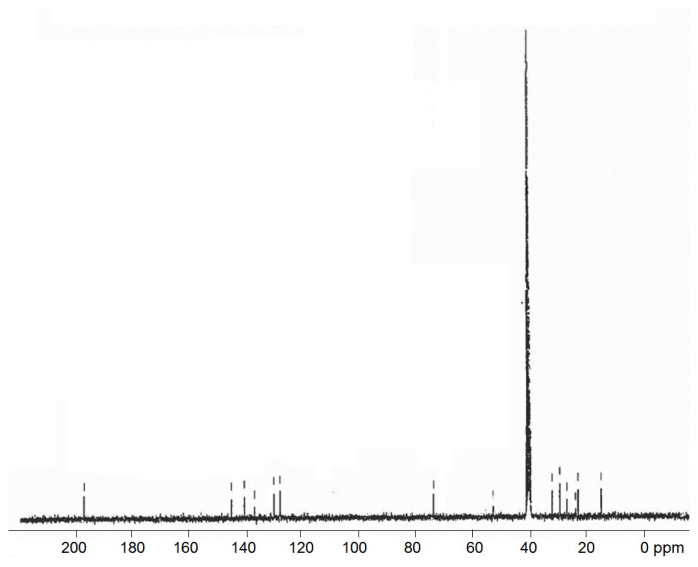
^13^C NMR spectrum of PA(C_8_)_3_ surfactant.

The number and types of groups of carbon atoms present in the compound are confirmed by ^13^C NMR spectroscopy and the predicted structure for the new surfactants can be justified by elaborating the NMR data. The spectrum of PA(C_8_)_3_ is shown in Figure 4. The characteristic shifts are illustrated in Table 5. The^13^C-NMR spectra of novel synthesized surfactants (PA(C_2_)_3_, PA(C_8_)_3_, PAC_16_ and PAC_18_) show characteristic chemical shifts: *δ* (ppm): 127–145, 127–140, 127–141 and 127–141.5 for aromatic carbon, 195.5, 196, 196.5 and 197 for (C=O), 70.5, 70, 69.8 and 68.5 for CH_2_, 60.5, 60, 58 and 59 for C-N, 21–32, 22–30.1, 22.8–29.7 for long chain carbon, 10.5, 14, 14.5 and 14 for CH_3_, respectively. These characteristic peaks confirm the formation of novel surfactants.

#### 3.2.5 FTIR spectroscopy

The FTIR spectrum was recorded for all four newly synthesized surfactants is given as: The peaks observed at (2917 and 2842 cm^−1^), (2915 and 2843 cm^−1^), (2919 cm^−1^ and 2846 cm^−1^) and (2918 and 2848 cm^−1^) corresponds to sp^3^ C-H stretch in IR spectra of PA(C_2_)_3_, PA(C_8_)_3_, PAC_16_ and PAC_18_, respectively. The vibrational band for (C=O) appears at 1681 cm^−1^, 1682 cm^−1^, 1682 cm^−1^ and 1683 cm^−1^, for (C=C) at 1604 cm^−1^, 1603 cm^−1^, 1601 cm^−1^, 1600 cm^−1^, for (C-N) at 1239 cm^−1^, 1237 cm^−1^, 1236 cm^−1^ and 1235 cm^−1^in the spectra of synthesized surfactants (PA(C_2_)_3_, PA(C_8_)_3_, PAC_16_ and PAC_18_), respectively. The CH_3_ and CH_2_ bending vibrations of PA(C_2_)_3_ appears at 1385 and 1463 cm^−1^, for PA(C_8_)_3_ at 1388 and 1467 cm^−1^, for PAC_16_ at 1384 and 1465 cm^−1^, for PAC_18_ at 1386 and 1462 cm^−1^. These peaks confirm the formation of novel synthesized surfactants. The FTIR data of newly synthesized surfactants is summed up in Table 6.

### 3.3 CMC values

The critical micelle concentration (CMC) i.e. the concentration of a surfactant in a solution at which micelle formation starts, decreases with the increase in chain length of the surfactants. The CMC of newly synthesized 4-phenyl phenacylbromide based surfactants has been determined using tensiometric method. Different dilutions were prepared from 1 mM stock solution of every surfactant and surface tension for each of these dilutions was recorded by a force tensiometer calibrated with distilled water. A graph was plotted between surface tension in nm^−1^ on y-axis and concentration in mM on x-axis, and CMC was identified as the intersecting point of two lines, the linear declining slope, and the baseline of minimal surface tension. The CMC plots for the new compounds are displayed in Table 7. Difference in hydrophobic chain length results in different CMC values [1,3 ]. Note that the carbon number in long chain of PA(C_8_)_3_ is the highest (24 carbons in 3 long chains), but the chain length is shorter that is why CMC value is high. Whereas for PAC_16_ and PAC_18_ surfactants the long chain carbons are lesser (16 and 18 carbons per chain) but the CMC value is lower as compared to PA(C_8_)_3_ surfactant. This makes the PAC_18_ surfactant most useful in terms of CMC.

### 3.4 Drug delivery

The drug delivery application of newly synthesized surfactants was carried out using the well-known anticancer drug cis-platin. The drug was encapsulated in PLGA nanoparticles along with surfactants. Surfactants act as encapsulating agents for the drug as well as stabilizers for nanoparticles which were lyophilized and stored for further study. Later the drug release was studied by suspending the resultant nanoparticles in phosphate buffer saline of pH 7.4.

#### 3.4.1 Drug delivery application

The initial CP concentration was taken as 5 mg. The drug encapsulation efficiency can be calculated by determination of encapsulated CP amount by using the following relation.


$$DEE=\frac{amount\;of\;CP\;in\;NPs}{initial\;mount\;of\;CP}\times 100$$

The drug loading content (DLC) can be calculate using the following relationship.


$$DLC=\frac{mass\;of\;CP\;in\;NPs}{mass\;of\;NPs}\times 100$$

The drug encapsulation efficiency for the prepared surfactants coated PLGA nanoparticle formulations range from 65%–67%, which are very similar to each other. The ease of micelle formation is depicted in the calculated encapsulation efficiency for the formulations prepared using surfactants. The drug loading content was found in the range of 1.96 to 2.31% and very similar for all NPs-surfactant formulations. The results shown above are also comparable to those of the already carried out experiments with cisplatin-PLGA supported with other compounds (mPEG etc) where DLC was found in the range of 1.99%–2.0% [20]. In a recent study [21] cisplatin-loaded PLGA NPs supported with chitosan shows the DLC of 6.67 ± 0.9% with an drug encapsulation efficiency of 62.99 ± 2.01%, for human epidermal growth factor receptor 2 targeted ovarian cancer therapy, similarly the other study shows the DLC of 3.9% and DEE of 72% for PLGA-mPEG NPs loaded with cisplatin [22]. The release of drug from cisplatin loaded PLGA-surfactant Nps was studied for 96 h and the results shows the release of drug was achieved up to 80.7 to 85.1% which better than the reported previously [22] where nearly 85 % was achieved in 120 h, similarly the other study shows the drug release of less than 40% in 70 h [21]. Similarly in another study [23], the percent loading of the PLGA-mPEG nanoparticles with cisplatin was shown to be significant (1%–2.5% w/w). In the proposed method, a modified double emulsion method was used to prepare PLGA-mPEG nanoparticles of cisplatin, which resulted in improved cisplatin loading in the PLGA-mPEG nanoparticles.

The drug encapsulation efficiency (DEE), LDC and drug release calculated for all nanoparticle formulations is listed in Table 8.

## Figures and Tables

**Table 1:** Composition of prepared nanoparticle.

Serial No.	Formulation	Surfactant	PLGA	Cis-platin
1.	PA(C_2_)_3_-PLGA	10 mg	100 mg	5 mg
2.	PA(C_8_)_3_-PLGA	10 mg	100 mg	5 mg
3.	PAC_16_-PLGA	10 mg	100 mg	5 mg
4.	PAC_18_-PLGA	10 mg	100 mg	5 mg

**Table 2:** Description of newly synthesized compounds.

S #	Reactants	Product			
	1st reactant	2nd reactant	Molecular formula	Structural formula	Percentage yield
1.	Phenyl-phenacyl bromide	Triethyl amine	C_20_H_26_ON	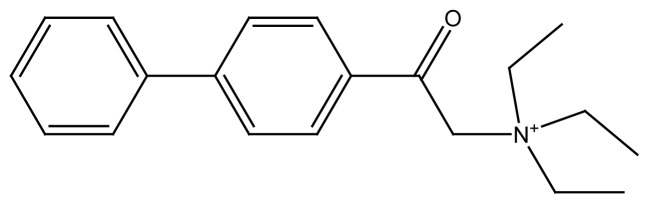	64.59%
2.	Phenyl-phenacyl bromide	Trioctyl amine	C_38_H_62_ON	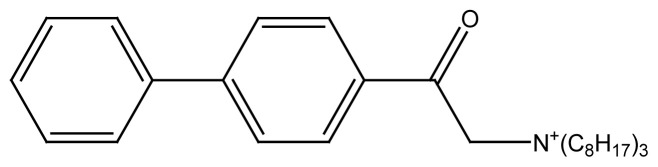	57.41%
3.	Phenyl-phenacyl bromide	Hexadecyl amine	C_30_H_44_ON	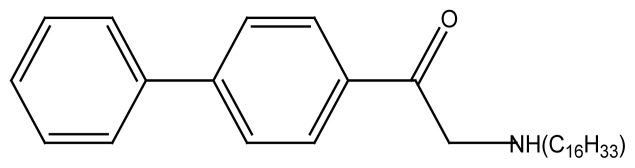	78.27%
4.	Phenyl-phenacyl bromide	Octadecyl amine	C_32_H_48_ON	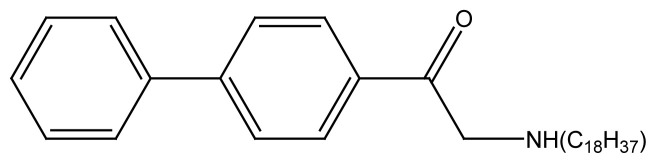	77.83%

**Table 3:** Physical characteristics of newly synthesized compounds.

Serial No.	Compound abbreviation	Molecular mass	Melting point	Solubility	Color
1.	PA(C_2_)_3_	296 g/mol	78°C	H_2_O,CHCl_3_, Acetone, DMSO,DMF	Light brown
2.	PA(C_8_)_3_	548 g/mol	156°C	H_2_O,CHCl_3_, Acetone, DMSO,DMF	Brown
3.	PAC_16_	434 g/mol	134°C	H_2_O,CHCl_3_, Acetone, DMSO,DMF	White
4.	PAC_18_	462 g/mol	139°C	H_2_O,CHCl_3_, Acetone, DMSO,DMF	White

**Table 4:** ^1^HNMR data of Newly synthesized compounds.

Bonding		PA(C_2_)_3_	PA(C_8_)_3_	PAC_16_	PAC_18_
Tail	Aromatic H	7.42–8.07 (m)	7.44–8.09(m)	7.42–7.92 (m)	7.42–8.02 (m)
	CH_2_	4.50 (s)	4.76 (s)	4.50 (s)	4.56 (s)
Head	CH_2_ -N	2.38 (q)	2.39 (t)	2.36 (m)	2.34 (m)
	Long chain	-	1.27 (m)	1.33 (m)	1.33 (m)
	CH_3_	1.25 (t)	0.90 (m)	0.96 (m)	0.97 (m)

**Table 5:** ^13^C NMR data of newly synthesized compounds.

Bonding		PA(C_2_)_3_	PA(C_8_)_3_	PAC_16_	PAC_18_
Tail	Aromatic C	127–143	127–145	127–141	127–141.5
	C = O	195.5	196	196.5	197
	CH_2_	72.5	73.5	72.8	72.5
Head	C-N	50.5	52.2	53	52.5
	Long chain C	-	22.5–31.6	22–30.1	22.8–29.7
	CH_3_	10.5	14.4	14.5	14

**Table 6:** FTIR data of newly synthesized compounds.

Bonding	PA(C_2_)_3_ (cm^−^^1^)	PA(C_8_)_3_ (cm^−^^1^)	PAC_16_ (cm^−^^1^)	PAC_18_ (cm^−^^1^)
C-N	1239	1237	1236	1235
C = O	1681	1682	1682	1683
C = C	1604	1603	1601	1600
CH_2_ bending	1463	1467	1465	1462
sp^3^ C-H stretch	2917, 2842	2915, 2843	2919, 2846	2918, 2848
CH_3_ bending	1385	1388	1384	1386

**Table 7:** CMC values of newly synthesized compounds.

S #	Surfactants	CMC (mM)
**1**.	**PA(C** **_2_** **)** **_3_**	**0.091**
**2**.	**PA(C** **_8_** **)** **_3_**	**0.028**
**3**.	**PAC** **_16_**	**0.026**
**4**.	**PAC** **_18_**	**0.024**

**Table 8:** Drug delivery parameters for cisplatin loaded PLGA-surfactant nanoparticles.

Serial No.	Nanoparticle formulation	Drug encapsulation efficiency (%)	Drug loading content (%)	Drug released (%)
1.	PA(C_2_)_3_-PLGA	65 ± 1.5	1.96 ± 0.11	85.1 ± 1.1
2.	PA(C_8_)_3_-PLGA	67 ± 1.6	2.12 ± 0.20	82.2 ± 2.1
3.	PAC_16_-PLGA	66 ± 1.8	2.07 ± 0.14	81.1 ± 1.7
4.	PAC_18_-PLGA	66 ± 2.1	2.31 ± 0.19	80.7 ± 1.9
